# Drastic Reductions in Mental Well-Being Observed Globally During the COVID-19 Pandemic: Results From the ASAP Survey

**DOI:** 10.3389/fmed.2021.578959

**Published:** 2021-03-26

**Authors:** Jan Wilke, Karsten Hollander, Lisa Mohr, Pascal Edouard, Chiara Fossati, Marcela González-Gross, Celso Sánchez Ramírez, Fernando Laiño, Benedict Tan, Julian David Pillay, Fabio Pigozzi, David Jimenez-Pavon, Matteo C. Sattler, Johannes Jaunig, Mandy Zhang, Mireille van Poppel, Christoph Heidt, Steffen Willwacher, Lutz Vogt, Evert Verhagen, Luiz Hespanhol, Adam S. Tenforde

**Affiliations:** ^1^Department of Sports Medicine, Goethe University Frankfurt, Frankfurt, Germany; ^2^Faculty of Medicine, Medical School Hamburg, Hamburg, Germany; ^3^Department of Physical Medicine and Rehabilitation, Spaulding Rehabilitation Hospital and Harvard Medical School, Charlestown, MA, United States; ^4^Inter-University Laboratory of Human Movement Science (LIBM EA 7424), University of Lyon, University Jean Monnet, Saint Etienne, France; ^5^Department of Clinical and Exercise Physiology, Sports Medicine Unity, Faculty of Medicine, University Hospital of Saint-Etienne, Saint-Etienne, France; ^6^Department of Movement, Human and Health Sciences, University of Rome “Foro Italico”, Rome, Italy; ^7^ImFine Research Group, Department of Health and Human Performance, Universidad Politécnica de Madrid, Madrid, Spain; ^8^Exercise is Medicine Spain, Madrid, Spain; ^9^School of Physical Activity Sciences, University of Santiago de Chile, Santiago, Chile; ^10^Fundación Instituto Superior de Ciencias de la Salud, Buenos Aires, Argentina; ^11^Department of Sport and Exercise Medicine, Changi General Hospital, Singapore, Singapore; ^12^Department of Basic Medical Sciences, Durban University of Technology, Durban, South Africa; ^13^MOVE-IT Research Group, Department of Physical Education, Faculty of Education Sciences, University of Cádiz, Cádiz, Spain; ^14^Institute of Human Movement Science, Sport and Health, University of Graz, Graz, Austria; ^15^Department of Orthopedics, University Children's Hospital Basel, University of Basel, Basel, Switzerland; ^16^School of Human Movement and Nutrition Sciences, The University of Queensland, Brisbane, QLD, Australia; ^17^Institute of Biomechanics and Orthopaedics, German Sport University Cologne, Cologne, Germany; ^18^Amsterdam Collaboration on Health and Safety in Sports, Department of Public and Occupational Health, Amsterdam Movement Sciences, Amsterdam UMC, University Medical Centers—Vrije Universiteit Amsterdam, Amsterdam, Netherlands; ^19^Masters and Doctoral Programs in Physical Therapy, Universidade Cidade de São Paulo (UNICID), São Paulo, Brazil

**Keywords:** coronavirus, WHO-5, SF-36, psychological health, pain, lockdowns

## Abstract

Most countries affected by the COVID-19 pandemic have repeatedly restricted public life to control the contagion. However, the health impact of confinement measures is hitherto unclear. We performed a multinational survey investigating changes in mental and physical well-being (MWB/PWB) during the first wave of the pandemic. A total of 14,975 individuals from 14 countries provided valid responses. Compared to pre-restrictions, MWB, as measured by the WHO-5 questionnaire, decreased considerably during restrictions (68.1 ± 16.9 to 51.9 ± 21.0 points). Whereas 14.2% of the participants met the cutoff for depression screening pre-restrictions, this share tripled to 45.2% during restrictions. Factors associated with clinically relevant decreases in MWB were female sex (odds ratio/OR = 1.20, 95% CI: 1.11–1.29), high physical activity levels pre-restrictions (OR = 1.29, 95% CI 1.16–1.42), decreased vigorous physical activity during restrictions (OR = 1.14, 95% CI: 1.05–1.23), and working (partially) outside the home vs. working remotely (OR = 1.29, 95% CI: 1.16–1.44/OR = 1.35, 95% CI: 1.23–1.47). Reductions, although smaller, were also seen for PWB. Scores in the SF-36 bodily pain subscale decreased from 85.8 ± 18.7% pre-restrictions to 81.3 ± 21.9% during restrictions. Clinically relevant decrements of PWB were associated with female sex (OR = 1.62, 95% CI: 1.50–1.75), high levels of public life restrictions (OR = 1.26, 95% CI: 1.18–1.36), and young age (OR = 1.10, 95% CI: 1.03–1.19). Study findings suggest lockdowns instituted during the COVID-19 pandemic may have had substantial adverse public health effects. The development of interventions mitigating losses in MWB and PWB is, thus, paramount when preparing for forthcoming waves of COVID-19 or future public life restrictions.

## Introduction

The pandemic associated with the novel coronavirus SARS-CoV2 (commonly referred to as COVID-19) has been managed using a variety of containment strategies. States with known cases instituted restrictions in public travel, school and business closures, stay-at-home orders, and quarantines. Despite their effectiveness in limiting virus transmission (1), lockdowns may have detrimental consequences for health. Even with no restrictions in place, social isolation results in a 29% higher mortality risk (2). Investigations of quarantine effects for previous pandemics (e.g., Ebola, MERS, and SARS) identified the occurrence of post-traumatic stress syndromes, confusion, anger, or symptoms of depression (3). In addition to reducing interpersonal contact, confinements rendered gyms, sports clubs, and public spaces inaccessible. This is of relevance because regular movement is associated with positive affect and life satisfaction (4). Furthermore, active individuals exhibit better nociceptive inhibition and have a lower risk of suffering from musculoskeletal disorders when compared with sedentary persons (5). In sum, it could be speculated that lockdowns cause decreases in both physical and mental well-being.

So far, the health impact of public life restrictions related to COVID-19 has mostly been examined in individual countries. For instance, reports from China (6, 7), Italy (8), and Greece (9) suggest considerable increases in anxiety and depression. As confinement measures affect an estimated minimum of 4 billion people worldwide (10), exploring changes in mental well-being on a multinational scale is an urgent need. The same applies to physical well-being. To the best of our knowledge, changes in the prevalence of musculoskeletal pain and related disability have not been studied. The present study, therefore, investigated the hypothesis that restricting public life to address the COVID-19 pandemic is globally associated with decreases in markers of psychological and physical health.

## Materials and Methods

### Ethics and Design

We report data from the Activity and Health during the SArs-CoV2 Pandemic (ASAP) survey (11), which was performed in April and May 2020. Ethics approval was obtained in each of the involved 14 countries (Australia, Austria, Argentina, Brazil, Chile, France, Germany, Italy, Netherlands, South Africa, Singapore, Switzerland, Spain, and the United States). Participants were 18 and older from countries with (1) official cases of SARS-CoV2 and (2) confinement measures limiting movement in public spaces. Recruitment strategies included social media promotion, mailing lists, and health-related organizations.

### Assessment

The well-being section of the ASAP questionnaire consisted of three parts. The first used a Likert scale to gauge the overall impact of public life restrictions on (a) mental and (b) physical well-being. In the second part, mental well-being was assessed by means of the World Health Organization Well-Being Index (WHO-5) questionnaire. It retrospectively measures agreement with five statements (feeling cheerful and in good spirits, feeling calm and relaxed, feeling active and vigorous, waking up feeling fresh and relaxed, having a daily life being filled with things of interest). Each item is answered on a Likert scale (0 = at no time, 1 = some of the time, 2 = less than half of the time, 3 = more than half of the time, 4 = most of the time, 5 = all of the time). A total score is calculated by multiplying the sum of all item values by four. The instrument is available in multiple languages and has high reliability and validity as a screening tool for depression (12): A sum score of ≤50 has been shown to exhibit 86% sensitivity and 81% specificity for a “screening diagnosis” of depression (12). The WHO-5 was answered twice, once referring to a typical period before public life restrictions and once referring to the time during restrictions.

In the third part, physical well-being was measured using the bodily pain subscale of the SF-36 questionnaire (SF-36 BPS). The instrument asks two questions assessing musculoskeletal pain (6-point Likert scale from “none” to “very severe”) and the resulting disability (5-point Likert scale from “not at all” to “extreme”). For the composite score, the average of both items is calculated and translated to a 0–100 scale. The SF-36 BPS is cross-culturally adapted and has both high internal consistency and reliability (13). To complement the results from the SF-36 BPS, we examined locations of musculoskeletal pain by adapting a checklist from a consensus statement on the reporting of epidemiological injury data (14). Also, the SF-36 BPS and the pain location checklist were completed twice: once for the time period preceding and once for the period during public life restrictions.

In addition to the background variables assessed in the ASAP questionnaire (sex, age, physical activity, work mode, and work volume), the level of national public life restrictions during the assessment period was quantified by means of the Containment and Health Index (15). The instrument systematically evaluates the governmental measures taken to contain viral spread (e.g., business closures, contact restrictions/tracing). The resulting score ranges between 1 and 100 with higher values representing stronger restrictions.

### Data Processing and Statistics

We conducted wave analyses to estimate the risk of nonresponse bias (16). Then, first, well-being changes from pre- to during restrictions were examined using Wilcoxon tests (Likert ratings of physical/mental well-being, subdimensions of the WHO-5 index) and paired *t*-tests for dependent samples (WHO-5 and SF-36 BPS scores), respectively. For the WHO-5, in addition to the sum score, the portion of participants below the cutoff for depression screening (≤50) pre- and during restrictions was determined.

In a second step, binary logistic regression (dependent variables: clinically relevant WHO-5 decrease of ≥10 (12) or minimally important SF-36 BPS decrease of ≥10 [13]) was used to calculate adjusted odds ratios (OR) including 95% confidence intervals for variables potentially moderating reductions in well-being. All data analyses were performed using SPSS 22 (SPSS Inc., Armonk, NY, USA). The significance level was set to α = 0.05.

## Results

Our sample consisted of 14,975 participants (38 ± 15 years, 58.1% females) from 14 countries. Levels of national public life restrictions were highest in Argentina, South Africa, and France and lowest in Brazil, Australia, and Switzerland (Table 1). Wave analyses yielded no indication of nonresponse bias (*p* < 0.05).

**Table 1:** Strength of governmental public life restrictions in the included countries as measured with the Containment and Health Index.

**Country**	**Containment and Health Index**
Australia	65.6 ± 1.5
Austria	79.2 ± 6
Argentina	88.5 ± 5.3
Brazil	63.3 ± 1.9
Chile	75.1 ± 0.5
France	81.8 ± 0
Germany	68.1 ± 2.4
Italy	87.1 ± 9.2
Netherlands	72.7 ± 0
South Africa	87.1 ± 0
Singapore	80.4 ± 9.8
Switzerland	68.3 ± 2.9
Spain	77.3 ± 1.6
United States	73.6 ± 0.7

*The table lists mean percentage values and standard deviation of changes during the study period. Higher values represent more restrictive measures*.

### Changes in Mental Well-Being

**Figure 1:**
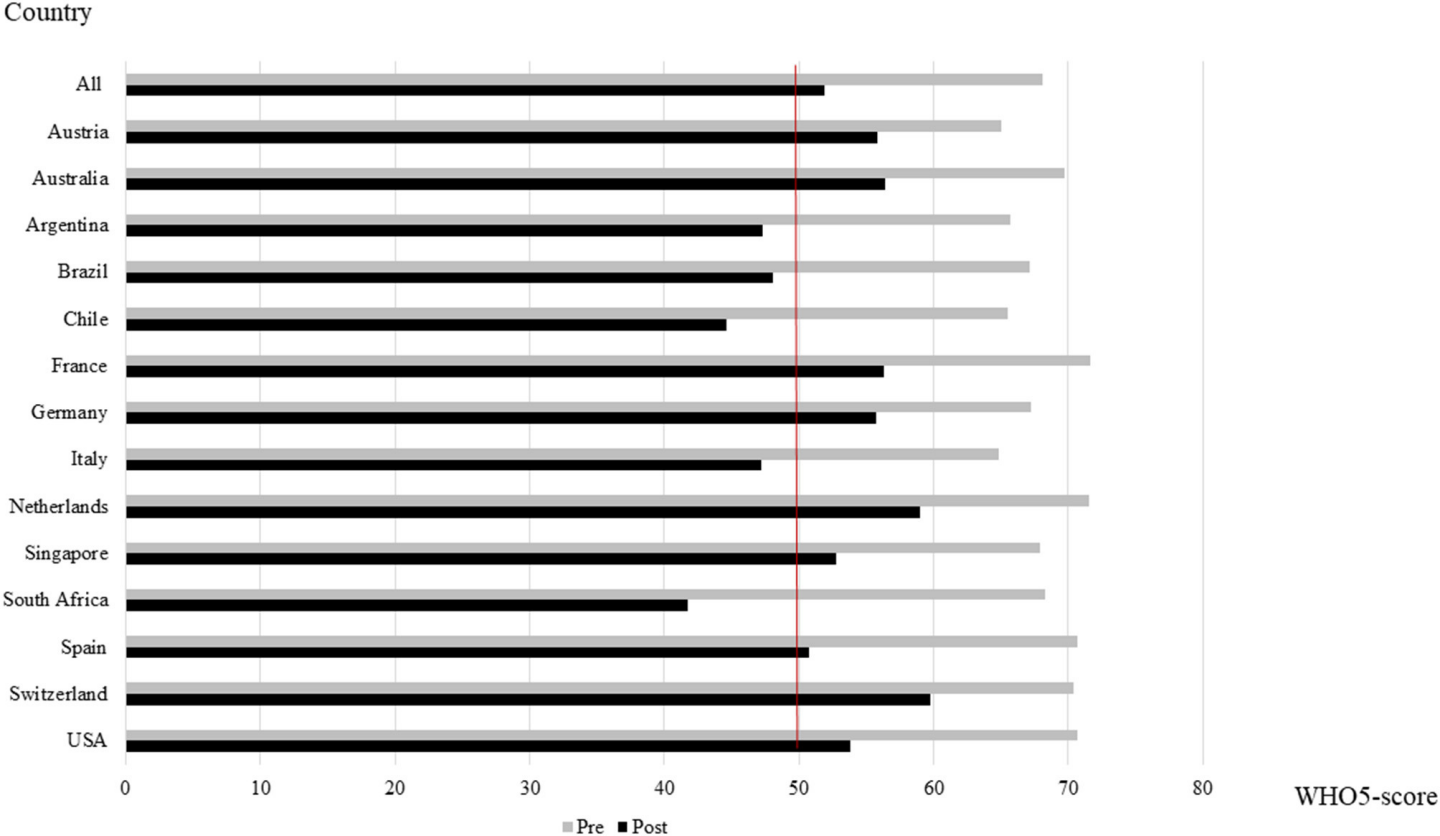
Mental well-being (mean WHO-5 sum score) pre- and during public life restrictions in all included countries. The red line indicates the cutoff score for depression screening (50%).

On the Likert scale, 73.0% (*n* = 10,916) of the participants reported a reduction in overall mental well-being although an improvement was indicated by 14.2% (*n* = 2130). Like the global rating (*p* < 0.001), also the WHO-5 score declined significantly from 68.1 ± 16.9 to 51.9 ± 21.0 during restrictions (*p* < 0.001, Figure 1). In the vast majority of cases (80.6%), the observed reductions were clinically relevant. Decreases were found on all items of the WHO-5 with highest reductions in “feeling active and vigorous” and “having a life filled with interesting things” (*p* < 0.001, Table 2). Pre-restrictions, 14.2% (*n* = 2,133) of participants met the cutoff for depression screening. This portion increased to 45.2% (*n* = 6,765) during restrictions.

**Table 2:** Subdimensions of the WHO-5 index before and during public life restrictions (median and interquartile range).

**WHO-5 item**	**Pre-restrictions**	**During restrictions**	**Wilcoxon test of difference**
I have felt cheerful in good spirits	4 (1)	3 (2)	*p* < 0.0001, *r* = −0.61
I have felt calm and relaxed	4 (1)	3 (2)	*p* < 0.0001, *r* = −0.32
I have felt active and vigorous	4 (1)	2 (2)	*p* < 0.0001, *r* = −0.56
I woke up feeling fresh and rested	3 (2)	3 (3)	*p* < 0.0001, *r* = −0.25
My life has been filled with things that interest me	4 (1)	2 (2)	*p* < 0.0001, *r* = −0.59

*The 6-point Likert scale has the following values: 0 = at no time, 1 = some of the time, 2 = less than half of the time, 3 = more than half of the time, 4 = most of the time, 5 = all of the time*.

Clinically relevant reductions of the WHO-5 score were associated with high physical activity levels pre-restrictions (OR = 1.29, 95% CI: 1.16–1.42), decreased vigorous physical activity during restrictions (OR = 1.14, 95% CI: 1.05–1.23), female sex (OR = 1.20, 95% CI: 1.11–1.29), working outside the home vs. working remotely (OR = 1.29, 95% CI: 1.16–1.44), and the combination of both vs. working remotely (OR = 1.35, 95% CI: 1.23–1.47). No associations were found for work volume (*p* = 0.42), age (*p* = 0.27), level of national public restrictions (*p* = 0.54), and changes in total physical activity during restrictions (*p* = 0.77).

### Changes in Physical Well-Being

**Figure 2:**
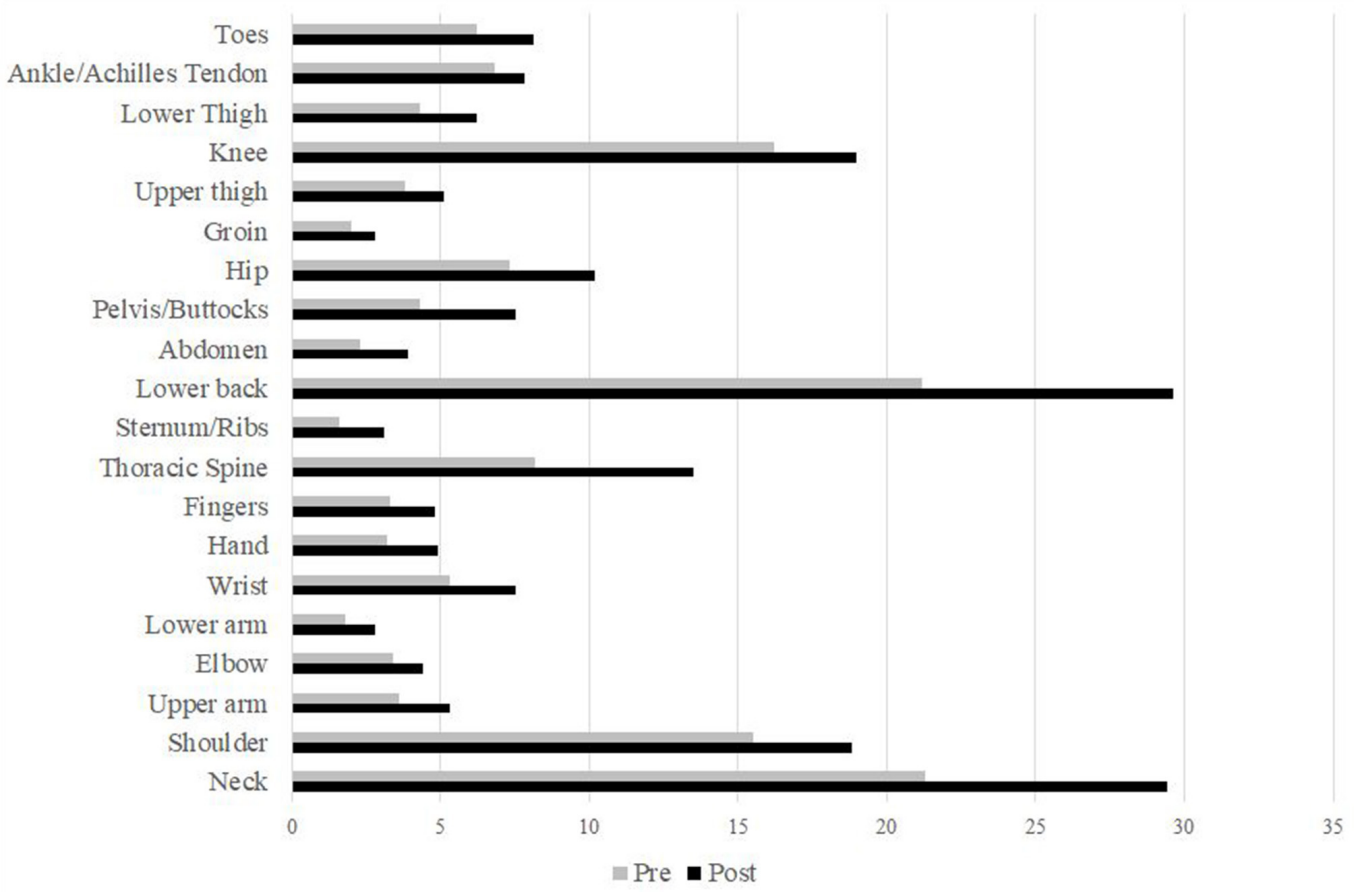
Prevalence [%] of musculoskeletal pain pre- and during public life restrictions stratified by body locations.

Almost two thirds (64.2%; *n* = 9,594) of the participants reported a reduction in overall physical well-being although an improvement was indicated by 20.0% (*n* = 2,985) of the surveyed individuals. Values on the SF-36 BPS decreased from 85.8 ± 18.7 to 81.3 ± 21.9% (*p* < 0.001). Regarding individual items, score reductions were higher for musculoskeletal pain (−7.1%) than for resulting disability (−3.8%). Prevalence of pain (Figure 2) increased in all body locations with the highest increments in the lower back (+8.4%), neck (+8.1%), and thoracic spine (+5.3%).

Clinically relevant decrements in physical well-being were associated with female sex (OR = 1.62, 95% CI: 1.50–1.75), high levels of national public life restrictions (OR = 1.26, 95% CI: 1.18–1.36), and young age (OR = 1.10, 95% CI: 1.03–1.19). No associations were found for work mode (*p* = 0.76), work volume (*p* = 0.10), pre-restriction physical activity level (*p* = 0.23), or moderate (*p* = 0.90) and vigorous physical activity (*p* = 0.22) during restrictions.

## Discussion

To the best of our knowledge, this is the first large-scale multinational investigation of physical and mental well-being during the first wave of the COVID-19 pandemic. Although confinement strategies seem effective in curbing the spread of the virus, they may entail a series of adverse health consequences. Most notably, the number of individuals at risk for depression has tripled during lockdowns. With almost half of our sample then falling below the screening cutoff, the reductions in mental well-being, observed across 14 countries, validate and expand available data from other pandemics (3) and early COVID-19 reports [e.g., (6–10)].

Impairments were smaller for physical than for mental well-being. In the first place, this may mean that interventions aiming to mitigate negative health consequences associated with lockdowns should particularly emphasize psychological aspects. However, careful consideration is still needed when aiming to interpret the seemingly low decreases in physical health. The SF-36 BPS combines ratings on musculoskeletal pain and disability. Most theoretical models assume that pain needs to be maintained for a certain period of time until disability manifests (17). Moreover, disability is significantly moderated by psychological distress and fear (17). As the survey referred to a period of a few weeks and as impairments in mental well-being were strong, a more pronounced increase in pain and dysfunction would be plausible at a later point in time.

Our results represent a call to action for health providers and policy makers. Impaired psychological well-being increases not only the odds of depression but also mortality risk (18). This highlights the importance of recognizing the negative mental health consequences during pandemic-related confinements. Newly developed interventions should specifically address the needs of women, who had higher odds for clinically relevant reductions in both the WHO-5 and the SF-36 BPS. Regarding psychological well-being, the present study's findings align with a wealth of evidence demonstrating a gender gap with a higher depression susceptibility of females (19). In summary, health stakeholders need to be aware that restrictions in public life may be associated with substantial decrements in mental and physical well-being. Interestingly, we found only a modest relationship between the restriction level and lower SF-36 values and no relationship between the restriction level and the WHO-5 scores. This may be because the Containment and Health Index contains several elements that are not directly related to individual well-being (e.g., contact tracing and testing paradigm).

Two strategies seem of value to mitigate losses in physical and, particularly, mental well-being. Existing literature suggests working remotely is associated with lower stress levels and decreased risk of depression (20, 21). Also, our survey revealed smaller odds for mental well-being reductions in persons working from home. Besides encouraging and allowing employees to change the workplace, the promotion of regular physical activity could be helpful. Reductions in vigorous activity during restrictions and a high baseline activity prior to restrictions were both related to declines in WHO-5 scores. This means that (a) having been active prepandemic is not protective against well-being decrements and (b) that the development of strategies aiming to maintain the previous movement habits is urgently needed.

Our survey provides strong indications of a subjective well-being decrease in the vast majority of participants. However, it is of interest that a substantial proportion also displayed improvements: One in seven individuals reported increased mental health, and one in five reported improved physical health. Possible reasons for this may include a variety of factors, such as higher amounts of time spent with family, higher task autonomy, reduced work-related travel, or reevaluation of personal health priorities.

Finally, some methodological aspects merit consideration. Our cross-sectional study used retrospective questions. It has been shown that self-reports of health outcomes may be affected by recall bias if relating to the past (22). Although we used relatively short time periods (days to weeks), this phenomenon cannot be ruled out entirely in the examined sample. Another issue relates to the influence and control of background variables: Although we assessed many factors, including age, sex, physical activity levels, work mode, and work volume, it would, inter alia, have been valuable to collect additional sociodemographic data, such as education, profession, and income.

## Conclusions

Confinements in countries affected by the novel coronavirus may have caused major reductions in subjective well-being. Strategies promoting telecommuting and maintenance of physical activity may help prevent similar losses in future pandemics or forthcoming waves of COVID-19.

## Data Availability Statement

The datasets presented in this article are not readily available because only aggregated data can be shared as per ethics approval. Requests to access the datasets should be directed to Jan Wilke, wilke@sport.uni-frankfurt.de.

## Ethics Statement

The study was approved by the Ethics committee of the Faculty of Psychology and Sports Sciences, Goethe University. The participants provided informed consent to participate in this study.

## Author Contributions

JW: concept/design, data collection, analysis, interpretation, writing, and critical revision. KH, LM, and AT: concept/design, data collection, analysis, interpretation, and critical revision. PE, CF, MG-G, CS, FL, BT, JP, FP, DJ-P, MS, JJ, MZ, MP, CH, SW, LV, EV, and LH: data collection, interpretation, writing, and critical revision. All authors contributed to the article and approved the submitted version.

## Conflict of Interest

The authors declare that the research was conducted in the absence of any commercial or financial relationships that could be construed as a potential conflict of interest.
